# Analysis of *ACE2* Genetic Variability among Populations Highlights a Possible Link with COVID-19-Related Neurological Complications

**DOI:** 10.3390/genes11070741

**Published:** 2020-07-03

**Authors:** Claudia Strafella, Valerio Caputo, Andrea Termine, Shila Barati, Stefano Gambardella, Paola Borgiani, Carlo Caltagirone, Giuseppe Novelli, Emiliano Giardina, Raffaella Cascella

**Affiliations:** 1 Medical Genetics Laboratory, Department of Biomedicine and Prevention, Tor Vergata University, 00133 Rome, Italy; v.caputo91@gmail.com (V.C.); borgiani@med.uniroma2.it (P.B.); gnovelli@me.com (G.N.); emiliano.giardina@uniroma2.it (E.G.); raffaella.cascella@uniroma2.it (R.C.); 2 Genomic Medicine Laboratory UILDM, IRCCS Santa Lucia Foundation, 00179 Rome, Italy; andreatermine544@gmail.com (A.T.); shila.barati@hotmail.it (S.B.); 3 Neuromed Institute IRCCS, 86077 Pozzilli, Italy; stefanogambardella@gmail.com; 4 Department of Biomolecular Sciences, University of Urbino “Carlo Bo”, 61029 Urbino, Italy; 5 Department of Clinical and Behavioral Neurology, IRCCS Fondazione Santa Lucia, 00179 Rome, Italy; c.caltagirone@hsantalucia.it; 6 Department of Biomedical Sciences, Catholic University Our Lady of Good Counsel, 1000 Tirana, Albania

**Keywords:** *ACE2* gene, SARS-Cov-2, COVID-19, eQTLs, bioinformatic analysis, neurological symptoms

## Abstract

Angiotensin-converting enzyme 2 (ACE2) has been recognized as the entry receptor of the novel severe acute respiratory syndrome coronavirus 2 (SARS-Cov-2). Structural and sequence variants in *ACE2* gene may affect its expression in different tissues and determine a differential response to SARS-Cov-2 infection and the COVID-19-related phenotype. The present study investigated the genetic variability of *ACE2* in terms of single nucleotide variants (SNVs), copy number variations (CNVs), and expression quantitative loci (eQTLs) in a cohort of 268 individuals representative of the general Italian population. The analysis identified five SNVs (rs35803318, rs41303171, rs774469453, rs773676270, and rs2285666) in the Italian cohort. Of them, rs35803318 and rs2285666 displayed a significant different frequency distribution in the Italian population with respect to worldwide population. The eQTLs analysis located in and targeting *ACE2* revealed a high distribution of eQTL variants in different brain tissues, suggesting a possible link between *ACE2* genetic variability and the neurological complications in patients with COVID-19. Further research is needed to clarify the possible relationship between *ACE2* expression and the susceptibility to neurological complications in patients with COVID-19. In fact, patients at higher risk of neurological involvement may need different monitoring and treatment strategies in order to prevent severe, permanent brain injury.

## 1. Introduction

Angiotensin-converting enzyme 2 (ACE2) has recently caught the attention of the scientific community, since it has been recognized as the entry receptor of the novel pathogenic severe acute respiratory syndrome coronavirus 2 (SARS-Cov-2) [1]. ACE2 is a protein encoded by its homologous gene (*ACE2*), which maps on chromosome X (Xp2.22) and consists of 18 exons. ACE2 is classified as an ectoenzyme with an extracellular catalytic domain, which is able to cleave circulating peptides [2]. ACE2 is expressed in several tissues, including airway epithelia, small intestine, heart, kidney, lung, testis, and brain [2,3]. Concerning the function of ACE2, it takes part in the renin angiotensin system (RAS) by converting Ang I to Ang-(1–9) and Ang II to Ang-(1–7) peptides, respectively. The function of Ang-(1–9) peptides is still unknown, whereas Ang-(1–7) peptides act as vasodilators. Moreover, ACE2 is able to cleave other peptides, including apelin, kinins, and morphins [2]. By cleavage and subsequent inactivation of angiotensin II, ACE2 has been recognized as an essential determinant in the regulation of blood pressure, vascular function, metabolism, and in the protection against atherosclerosis, heart, kidney, brain, and lung injuries [2,3]. However, a pathophysiological function has also be assigned to ACE2, which is as the entry receptor for SARS-Cov-1 and the novel SARS-Cov-2 viruses, which have been responsible for the sever acute respiratory syndrome (SARS) epidemic in 2003 and the current coronavirus infectious disease (COVID-19) pandemic, respectively [1]. Thanks to high binding affinity between the viral spike (S) protein and ACE2 receptor, SARS-Cov-2 is able to enter into the human cells, and hijacks the cellular mechanisms for replicating itself and invading other cells [4]. Although ACE2 is utilized by SARS-Cov-2 as entry receptor, an accessory protein is necessary to prime the S protein, which is the transmembrane serine protease 2 (TMPRSS2) that is highly expressed in tissues of the respiratory tract, including the bronchus, pharyngeal mucosa, and lung, and is known to be critical for viral spread and pathogenesis in the infected host [4,5,6]. The lung is the most affected organ by SARS-Cov-2 infection, which has been shown to cause severe cytopathic effects in infected lung alveolar cells and induce an acute immuno-inflammatory response that, ultimately, results in lung injury [7]. It is important to note that ACE2 expression normally protects from lung injury and has been found to be downregulated upon SARS-Cov-1 infection [4]. ACE2 downregulation has been regarded as an innate immune defense mechanism to impair viral entry into the host cells [8]. On the other hand, the downregulation of ACE2 has also been shown to be a mechanism induced by the virus to fasten its cell-to-cell spreading by triggering the injury of the host lung tissue [8]. On this subject, whether SARS-Cov-2 interferes with ACE2 expression has yet to be determined, although it is an interesting matter of investigation. The SARS-Cov-2 infection resulting in COVID-19 symptomatology is characterized by a range of symptoms, including dry cough, fever, myalgia or fatigue, dyspnoea, and pneumonia [1,9]. In the most severe cases, several clinical complications (acute respiratory distress syndrome, sepsis, acute cardiac injury, acute kidney injury, and secondary infections) have been described and associated with a more systemic response to the infection, the results of which can be fatal [9,10]. In addition to systemic and respiratory symptoms, 14–36% of severe patients with COVID-19 show neurological signs, including dizziness, headache, taste and smell impairment, impaired consciousness, encephalitis, seizures, ataxia, and stroke [11,12]. This finding, together with the evidence of SARS-Cov-2 in the cerebrospinal fluid of patients with COVID-19, suggested that the neurotropic effects of SARS-Cov-2 may contribute to the morbidity and mortality caused by COVID-19 [3]. Given the multiple roles of ACE2 and its expression pattern in different tissues, the alteration of its expression levels may have an impact on the susceptibility, symptomatology, and outcome of COVID-19. In particular, the presence of structural and sequence variants in the *ACE2* gene may affect its expression in different tissues and determine a differential response to the SARS-Cov-2 infection. In this context, the present study aimed to investigate the genetic variability of *ACE2* in terms of single nucleotide variants (SNVs), copy number variations (CNVs) and expression quantitative loci (eQTLs) in a cohort of 268 Italian individuals.

## 2. Materials and Methods 

The study was performed by utilizing 268 DNA samples representative of the Italian general population, which were partially available at the Genomic Medicine Laboratory of Santa Lucia Foundation Hospital and partially derived from international databases. The Italian cohort of samples was composed of 100 samples analyzed by a comparative genomic hybridization (aCGH) array for assessing the presence of structural genomic variations, and 168 samples utilized for identifying common and rare variants located in the coding or splice site regions of the genome. The genetic data referred to these samples were partially derived by whole exome sequencing (WES), available at the Genomic Medicine Laboratory of IRCCS Santa Lucia Foundation Hospital and partially extracted by the Ensembl database [13,14,15]. Italian patients had an average age of 46 ± 15 years and a female/male ratio of 45:55. The research was approved by the Ethics Committee of Santa Lucia Foundation of Rome (CE/PROG.650, approved on 1 March 2018), and was performed according to the Declaration of Helsinki. The participants provided signed informed consent. 

The CNV analysis was performed by Chromosome Analysis Suite (ChAS) 3.1 (Affymetrix, Santa Clara, CA, USA) using the Cytoscan750k_Array Single Sample analysis “NA33_hg19” as reference file and an average resolution of 100 Kb.

**Figure 1:**
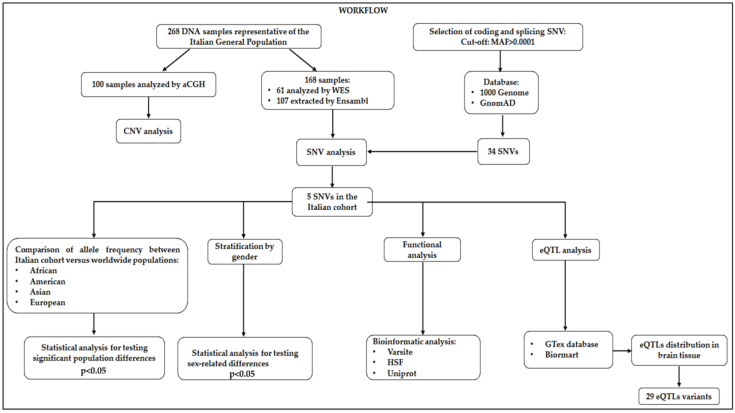
Workflow of the analytical steps performed in the study. CNV: copy number variation; SNV: single nucleotide variants; MAF: minor allele frequency; HSF: human splicing finder, eQTL: expression quantitative locus.

Concerning SNVs, we decided to analyze SNVs (namely SNPs and indels) located within the coding and splice site DNA regions, because they are most likely to affect protein function. We therefore selected the SNVs of interest by extracting the variants localized in the exonic and splice site regions of *ACE2*, whose frequency data were available from the 1000 Genomes and GnomAD databases [15,16,17,18]. The frequency cutoff for selecting the genetic variants of interest was set at minor allele frequency (MAF) > 0.0001. This approach allowed for selecting 34 putative variants located within the *ACE2* sequence (Table S1). The Ensembl database [15] was also utilized to download the allele frequency data of the 34 SNVs of interest in the worldwide populations, in order to compare the frequencies between the Italian cohort and the frequencies observed in African, American, Asian, and European populations. The presence of the 34 SNVs in the Italian samples was evaluated by analyzing the output file derived by the WES and Ensembl databases. For WES results, a coverage of 20X was considered for the analysis of *ACE2* sequence. The variant caller files (VCFs) obtained by WES analysis were first scanned with vcfR [19], and then subjected to analysis by “genomic variants filtering by deep learning models in NGS” (GARFIELD-NGS) [20]. In particular, vcfR is a package that enables visualization, manipulation, and performing quality control of VCF data [19]. GARFIELD-NGS is an informatics tool, which relies on deep learning models to dissect false and true variants in exome sequencing experiments [20]. The allelic frequency distribution of the detected SNVs and the existence of significant differences among Italian and worldwide populations were calculated by statistical tools. All statistical analyses were performed in an R environment [21]. A two-sided Fisher’s exact test and a *p*-value (*p*) were calculated in order to assess the different allelic distributions of detected SNVs in the Italian cohorts with respect to the other populations. The significance threshold was set at *p* < 0.05. In addition, multiple testing correction (false discovery rate) was performed by calculating the *q*-value (*q*) [22] and setting a significance cutoff at at *q* < 0.05. Considering that *ACE2* maps to the X chromosome, statistical analysis (two-sided Fisher’s exact test) was also performed by stratifying the cohorts according to gender to evaluate sex-related effects. Moreover, the SNVs detected in the Italian population were subjected to bioinformatic predictive analysis to assess their potential impact on ACE2 protein function and splicing mechanisms. To this purpose, VarSite [23], Human Splicing Finder (HSF) [24], and Uniprot database [25] were interrogated. VarSite analyzes and predicts the effect of amino acid changes on the protein structure. HSF evaluates the effects of variants on the splicing mechanisms. Moreover, Uniprot annotation database was utilized to retrieve the topological and functional domains organization of proteins. Concerning eQTL analysis, the Genotype-Tissue Expression (GTex) database [26] was utilized to retrieve the eQTLs variants with a significant effect on ACE2 expression in different tissues, and Biomart tool [27] was used to extract the significant eQTLs distributed on the basis of the affected tissue. GTex database is a public resource that enables us to study tissue-specific gene expression and regulation, as well as their relationship to genetic variation [26]. The significance threshold for eQTL analysis was set at *p* < 0.05. Biomart is a web-based tool that allows one to extract data in a uniform way and filter them for different queries [27]. In this study, Biomart was utilized to extract and filter the eQTLs significantly distributed in brain tissues. The entire analytical workflow of the study is illustrated in Figure 1. Moreover, all raw data utilized for statistical and computational analysis are available at the following link: https://github.com/Andreater/Data-and-RMD-for-ACE2-article.

## 3. Results and Discussion

**Figure 2:**
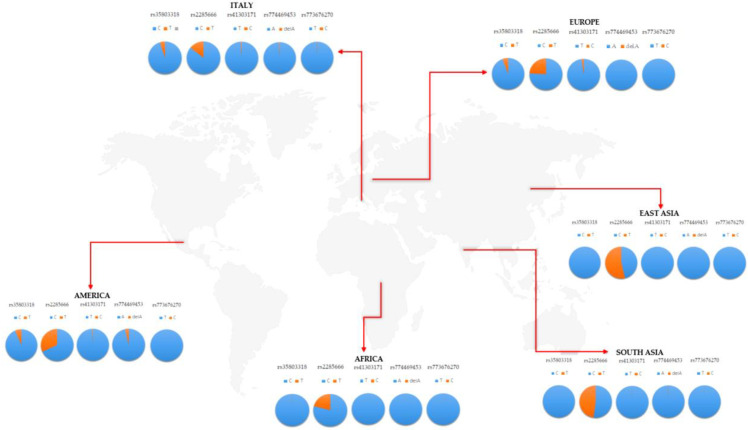
Frequency distribution of the five single nucleotide variants (SNVs) in the Italian cohort with respect to worldwide populations.

The final goal of the study has been the research of variants potentially affecting ACE2 expression and function, which may contribute to SARS-Cov-2 spreading among worldwide populations, and may have a clinical significance regarding the clinical variability and outcome displayed by patients with COVID-19. The analysis of CNVs in *ACE2* did not report any significant variation in our study cohort, ruling out that frequent copy number variations could potentially impact *ACE2* expression. Concerning SNVs, though, the screening of the 34 SNVs previously selected was performed on 168 Italian samples, and revealed the presence of five variants: rs35803318 (C/T), rs41303171 (T/C), rs774469453 (A/-), rs773676270 (T/C), and rs2285666 (C/T). These variants presented some differences in the frequency distribution in the Italian cohort, with respect to worldwide populations (Table 1 and Figure 2).

As expected, the Italian cohort showed overlapping frequency distributions with the European population, whereas significant differences were observed with respect to African, American, and Asian populations (Table 2). The statistical significance was also confirmed after correction for multiple testing (*q*-value) in these cohorts (Table 2).

In particular, the rs35803318 (C/T) is a synonymous variant, whose allelic frequency resulted in being significantly different from the frequencies observed in African and Asian populations, whereas it overlapped with frequencies recorded in the European and American groups (Table 1 and Table 2). In fact, the rs35803318 appeared to be more frequent in Italian, European, and American populations, compared to the very low frequency observed in the African and Asian cohorts (Figure 2). Concerning rs41303171, the Italian population showed overlapping frequencies with American, European, and Asian populations but not with the African cohort, in which the variant allele was almost absent (Figure 2). The frequency of rs774469453 in the Italian population overlaps with all investigated populations except for the American group, which reported a slightly higher frequency (Figure 2). The rs773676270 did not display significant differences in terms of frequency distribution of wild-type and variant alleles between the Italian and the other worldwide populations (Table 2). The variant allele was extremely rare among all populations, with a slight increase in the frequency observed in the Italian cohort (Table 1, Figure 2). However, none of these variants (rs41303171, rs774469453, and rs773676270) reported significantly different distributions among populations after correction for multiple testing (Table 2). Concerning rs2285666, this is the only variant showing a higher frequency, with respect to other variants previously discussed, that appeared to be very rare among the investigated populations (Figure 2). Concerning the frequency of rs2285666 in the Italian population, it was found to be significantly different with respect to the African, American, Asian, and even European population (Table 2). After multiple tests, the significance was maintained for American and Asian populations (Table 2). In particular, the variant allele of rs2285666 showed the lowest frequency in the Italian cohort compared to the other populations (Table 1, Figure 2). The frequency of the five SNVs were similarly distributed among male and female patients in each population, as well as between different populations, indicating that there are no gender effects underlying the frequency distribution of *ACE2* variants. Altogether, the analysis of the frequency distribution of *ACE2* coding variants in the Italian population with respect to worldwide populations showed a low rate of coding variants in *ACE2* gene in the Italian cohort, suggesting that the susceptibility to SARS-Cov-2 infection may depend from other genetic variants outside *ACE2*, or in other genes. On this subject, it would be interesting to investigate the possible contribution of non-coding variants located in the regulatory regions (such as promoters and enhancers) of *ACE2* to the risk for SARS-Cov-2 infection. In addition, further research should be performed to assess possible population-specific effects that may explain the variable susceptibility to SARS-Cov-2 infection in the Italian as well as in the worldwide populations. Consistent with our findings, a similar study on the Chinese population investigated the genetic variability of *ACE2* in their population, finding a different frequency distribution of *ACE2* variants with respect to the other populations [28]. Moreover, they found a higher allelic frequency of eQTL variants, which is associated with higher *ACE2* expression in tissues, suggesting a different susceptibility or response to SARS-Cov-2 infection with respect to other populations under similar conditions [28]. However, these data are not sufficient to demonstrate direct evidence of a relationship between *ACE2* genetic variants and a differential susceptibility to SARS-Cov-2 infection among populations. In fact, data obtained on Italian and Chinese populations should be replicated in larger cohorts and in case/control studies, in order to assess their potential association with susceptibility to SARS-Cov-2 infection. Moreover, further functional studies should be carried out to demonstrate and explain these eventual associations.

Concerning the functional analysis of the five SNV variants identified in the Italian cohort, most results were inconclusive or not significant to predict the functional impact of such variants on the resulting proteins. The rs35803318 is a synonymous variant, so it does not result in an amino acid change, and thus it is unlikely to affect the protein function. Moreover, it is located in a region coding for the transmembrane portion of the protein, which normally does not interact with the SARS-Cov-2 S protein. The rs41303171 is a missense variant, resulting in the amino acid change from asparagine (Asn) with a neutral side chain to aspartate carrying a negatively charged side chain, which is therefore more hydrophilic. However, interrogation of Varsite showed that the change from Asn to Asp is not a large one, indicating that it may or may not result in a change to the protein’s function. The prediction analysis of the impact of rs41303171 on protein function was therefore inconclusive, so it is not possible to predict the functional impact of this variant at the moment. However, it is interesting to note that the rs41303171 is localized in the region coding for the extracellular portion of ACE2, which normally interacts with the SARS-Cov-2 S protein. It will be therefore interesting to evaluate the impact of this variant by functional experiments in future. The rs774469453 variant is a single nucleotide deletion and is located in a splicing intronic region. Therefore, it has been subjected to HSF analysis, in order to test the variant for a potential alteration of splicing. The HSF interrogation showed that the variant allele of rs774469453 may create an exonic splicing silencer (ESS) site, but it is not significant, and therefore it probably does not affect splicing. The rs773676270 is a synonymous variant localized in a region encoding the extracellular portion of the protein, which interacts with the S protein of SARS-Cov-2. Interestingly, the interrogation of HSF reported that this variant may affect the splicing by activating an exonic cryptic acceptor site or altering an exonic splicing enhancer (ESE) site. These findings suggest that rs773676270 should be further investigated, together with other variants that have a larger effect on ACE2 function. The rs2285666 variant was a variant located in the splice site region of *ACE2*. However, the prediction analysis by HSF did not reveal significant splicing alterations.

**Figure 3:**
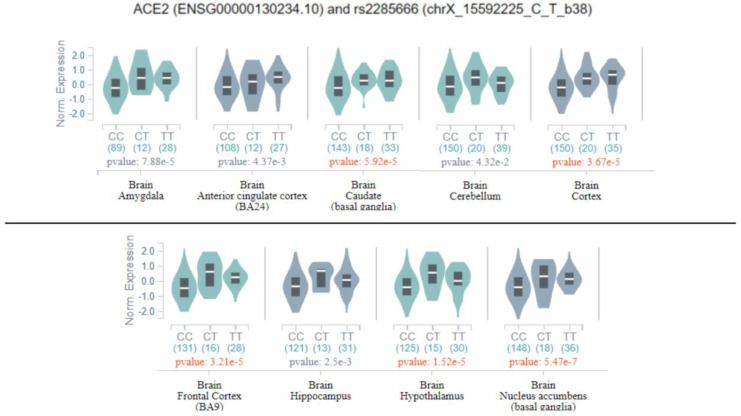
Violin plots represent the correlation between rs2285666 genotypes and *ACE2* mRNA expression in different brain tissues. The reports on the *x*-axis are the genotypes, with the correspondent counts in brackets. On the *y*-axis, the normalized expression of *ACE2* is reported. Moreover, a *p*-value of for the variant significance is reported. The figure has been obtained from Genotype-Tissue Expression (GTex) [26].

Successively, all previously discussed variants were evaluated as potential eQTLs in the GTEx database, and only rs2285666 was classified as a significant eQTL in several brain tissues, namely the amygdala, anterior cingulate cortex, basal ganglia, cortex, cerebellum, hippocampus, and hypothalamus (Figure 3).

As shown in Figure 3, the homozygous genotype for the variant allele may increase the expression of *ACE2* in multiple brain tissues, and consequently, may affect ACE2 functions in the brain. This finding suggests that the genetic variability of *ACE2* may have a greater impact on COVID-19-related symptoms and SARS-Cov-2 tissue tropism, rather than on the susceptibility to SARS-Cov-2 infection. On this subject, evidence of genetic variants in *TMPRSS* and a large presence of eQTLs in the lung may suggest that the genetic variability of *TMPRSS* might have a role in determining the different susceptibility to SARS-Cov-2 infection among populations. However, these are preliminary observations, which have to be confirmed by further investigations.

Considering the broader effect of rs2285666 in multiple brain tissues, we decided to look at the distribution of the eQTL variants located in and targeting *ACE2* in the different brain tissues, which may thereby affect *ACE2* expression, and in turn, contribute to the neurological symptoms and complications observed in patients with COVID-19. Interestingly, literature studies have highlighted the crucial role of ACE2 in brain physiology and pathophysiology, including marked regulatory effects on blood pressure, cardiac hypertrophy, stress response, anxiety, cognition, and brain injury [2,29,30]. The eQTL analysis on the GTex portal and Biomart allowed identification of 29 significant eQTLs, which have been predicted to affect *ACE2* expression in the brain at different levels (Table S2). Most of them (23 eQTLs) have a significant effect on multiple brain tissues, suggesting that they may affect ACE2-related brain functions as a whole. Six eQTLs instead showed a more tissue-specific effect, indicating that they may be involved in the alteration of brain functions regulated by restricted areas of the brain. Interestingly, the mostly enriched tissues with significant *ACE2*-associated eQTLs were the basal ganglia, cortex, hypothalamus, and substantia nigra, whereas the amygdala and cerebellum appeared to be less affected. These findings suggest that the alteration of *ACE2* expression may be involved in different neurological symptoms (seizures, stroke, encephalitis, dizziness, headache, confusion, alteration of body temperature, anosmia, and ataxia) observed in COVID-19 patients, in relation to the brain-affected area. Interestingly, none of *ACE2* eQTL variants located in and targeting *ACE2* were reported in the lung tissue, which, instead, appeared particularly enriched in *TMPRSS*-associated eQTL variants. These findings raise the need for further investigation on the role of *ACE2* genetic variability in the susceptibility and clinical outcome of patients with COVID-19, especially concerning neurological symptoms. Indeed, these studies will be useful for identifying patients at a higher risk of neurological complications, which may need different monitoring and treatment strategies in order to prevent fatal outcomes or severe, permanent brain injury.

## Figures and Tables

**Table 1:** Comparison of *ACE2* allelic variants between the Italian and worldwide populations. Allele counts of the variants of interest for each population are shown. ITA: 168 Italians subjects; AFR: 661 African subjects from 1000 Genomes; Americans: 446 subjects, composed of 99 CEU (Utah residents with Northern and Western European ancestry) subjects and 347 AMR (Latino Americans) subjects, from 1000 Genomes; Europeans: 297 subjects composed of 107 IBS (Iberian population in Spain) subjects, 91 GBR (British in England and Scotland), and 99 FIN (Finnish in Finland) subjects, from 1000 Genomes; EAS: 504 East Asian subjects from 1000 Genomes; SAS: 489 South Asian subjects from 1000 Genomes. ACs: allele counts. SNV: Single Nucleotide Variant

SNV	ITA	AFR	AMR	EUR	EAS	SAS
	ACs	ACs	ACs	ACs	ACs	ACs
**rs35803318 C/T**	C:231	C:1002	C:629	C:430	C:764	C:718
	T:13	T:1	T:44	T:26	T:0	T:0
**rs2285666 C/T**	C:209	C:791	C:458	C:345	C:354	C:374
	T:35	T:212	T:215	T:111	T:410	T:344
**rs41303171 T/C**	T:242	T:1003	T:668	T:447	T:764	T:717
	C:2	C:0	C:5	C:9	C:0	C:1
**rs774469453 A/-**	A:242	A:1002	A:650	A:456	A:757	A:716
	-:2	-:1	-:23	-:0	-:7	-:2
**rs773676270 T/C**	T:243	T:1003	T:673	T:456	T:764	T:717
	C:1	C:0	C:0	C:0	C:0	C:1

**Table 2:** Statistical results of the five SNVs in the Italian population versus (vs.) worldwide populations. Two-sided Fisher exact test’s *p*-value (*p*) showing the statistically significant differences of the frequency distributions of the five *ACE2* variants detected in the Italian cohort, with respect to the frequencies observed in the other populations. The *q*-value (*q*) show the adjusted *p* after multiple testing correction. The significant data are highlighted in bold. ITA: Italians; AFR: Africans; AMR: Americans; EUR: Europeans; EAS: East Asians; SAS: South Asians; ns: not significant.

SNP	*p*-Value ITA vs. AFR	*q*-Value ITA vs. AFR	*p*-Value ITA vs. AMR	*q*-Value ITA vs. AMR	*p*-Value ITA vs. EUR	*q*-Value ITA vs. EUR	*p*-Value ITA vs. EAS	*q*-Value ITA vs. EAS	*p*-Value ITA vs. SAS	*q*-Value ITA vs. SAS
**rs35803318 C/T**	**5.48 × 10** ^**9**^	**1.86 × 10** ^**7**^	ns	ns	ns	ns	**7.65 × 10** ^**9**^	**1.30 × 10** ^**7**^	**1.41 × 10** ^**8**^	**2.39 × 10** ^**7**^
**rs2285666 C/T**	0.016	0.267	**4.1 × 10** ^**8**^	**1.39 × 10** ^**6**^	0.002	0.06	**2.11 × 10** ^**29**^	**7.19 × 10** ^**8**^	**3.55 × 10** ^**22**^	**1.20 × 10** ^**20**^
**rs41303171 T/C**	0.038	0.432	ns	ns	ns	ns	ns	ns	ns	ns
**rs774469453 A/-**	ns	ns	0.036	0.62	ns	ns	ns	ns	ns	ns
**rs773676270 T/C**	ns	ns	ns	ns	ns	ns	ns	ns	ns	ns

## Supplementary Materials

The following supplementary files are available online at https://www.mdpi.com/2073-4425/11/7/741/s1, Table S1: Selection of the variants within the coding and splice site regions of *ACE2* with frequency data, annotated in 1000 Genomes. The reported variants were retrieved from Ensembl GRCh37 assembly. Chr: chromosome; bp: base pairs; MAF: Minor Allele Frequency; AA: amino acid; Table S2: List of the 29 significant eQTL variants distributed in relation to brain tissues.
