# Supplementary material for: Analysis of ACE2 Genetic Variability among Populations Highlights a Possible Link with COVID-19-Related Neurological Complications
Source: Genes (Basel). 2020 Jul 3;11(7):741. doi: 10.3390/genes11070741 (PMC7397291; doi:10.3390/genes11070741)
Supplement: Supplementary file 1 [file genes-11-00741-s001.zip › Table S1.docx]

**Supplementary Table 2**

**Analysis Of *ACE2* Genetic Variability Among Populations Highlights A Possible Link With Covid19-Related Neurological Complications**

Claudia Strafella^1,2,^ *, Valerio Caputo^1,2^, Andrea Termine^2^, Shila Barati^2^, Stefano Gambardella^3,4^, Paola Borgiani^1^, Carlo Caltagirone^5^, Giuseppe Novelli^1,3^, Emiliano Giardina^1,2, #^, Raffaella Cascella^1,6, #^

^1^Medical Genetics Laboratory, Department of Biomedicine and Prevention, Tor Vergata University, Rome, 00133, Italy

^2^Genomic Medicine Laboratory UILDM, IRCCS Santa Lucia Foundation, Rome, 00179, Italy

^3^Neuromed Institute IRCCS, Pozzilli, 86077, Italy

^4^Department of Biomolecular Sciences, University of Urbino "Carlo Bo", Urbino, 61029, Italy

^5^Department of Clinical and Behavioral Neurology, IRCCS Fondazione Santa Lucia, Rome, 00179, Italy

^6^Department of Biomedical Sciences, Catholic University Our Lady of Good Counsel, Tirana, 1000, Albania

# These authors equally contributed to the work.

*Corresponding author: claudia.strafella@gmail.com

**Table S2.** Selection of the variants within the coding and splice site regions of *ACE2* with frequency data annotated in 1000 Genomes. The reported variants were retrieved from Ensembl GRCh37 assembly. Chr: chromosome; bp: base pairs; MAF: Minor Allele Frequency; AA: amino acid.

| Variant ID | Chr: bp | Alleles | Global MAF | Variants Consequence | AA | AA coordinates |
| --- | --- | --- | --- | --- | --- | --- |
| rs35803318 | X:15582209 | C/T | T: 0.021 | synonymous | V | 749 |
| rs147311723 | X:15582265 | G/A | A: 0.005 | missense | L/F | 731 |
| rs41303171 | X:15582298 | T/C | C: 0.005 | missense | N/D | 720 |
| rs149039346 | X:15584416 | A/G | G: 0.001 | missense | S/P | 692 |
| rs777410473 | X:15584425 | T/C | C: < 0.001 | missense | K/E | 689 |
| rs200180615 | X:15584488 | C/T | T: < 0.001 | missense | E/K | 668 |
| rs199951323 | X:15585879 | A/C | C: < 0.001 | stop gained | L/* | 656 |
| rs183135788 | X:15585933 | T/C | C: < 0.001 | missense | N/S | 638 |
| rs774469453 | X:15589926 | A/- | -: 0.009 | splice region ~intron |  |  |
| rs756905974 | X:15590351 | T/C | C: < 0.001 | missense | N/S | 546 |
| rs202137736 | X:15591485 | T/C | C: < 0.001 | splice region ~intron |  |  |
| rs779199005 | X:15591503 | A/G | G: < 0.001 | missense | Y/H | 510 |
| rs748701949 | X:15591522 | C/T | T: < 0.001 | synonymous | L | 503 |
| rs140473595 | X:15591530 | C/T | T: < 0.001 | missense | A/T | 501 |
| rs200973492 | X:15591568 | A/G | G: < 0.001 | missense | V/A | 488 |
| rs191860450 | X:15593829 | T/C | C: < 0.001 | missense | I/V | 468 |
| rs199569050 | X:15593920 | G/A | A: < 0.001 | synonymous | N | 437 |
| rs773676270 | X:15596345 | T/C | C: < 0.001 | synonymous | Q | 388 |
| rs147464721 | X:15599363 | G/A | A: 0.002 | synonymous | L | 351 |
| rs138390800 | X:15599392 | T/C | C: < 0.001 | missense | K/R | 341 |
| rs185525294 | X:15599420 | T/A | A: < 0.001 | missense | M/L | 332 |
| rs780574871 | X:15599480 | C/T | T: < 0.001 | missense | E/K | 312 |
| rs749750821 | X:15599507 | C/T | T: < 0.001 | missense | D/N | 303 |
| rs745514718 | X:15605908 | C/T | T: < 0.001 | missense | S/N | 257 |
| rs372272603 | X:15607508 | G/A | A: < 0.001 | missense | R/C | 219 |
| rs779790336 | X:15607552 | C/G | G: < 0.001 | missense | R/T | 204 |
| rs2285666 | X:15610348 | C/T | T: 0.350 | splice region ~intron |  |  |
| rs528054982 | X:15610452 | A/T | T: < 0.001 | splice region ~intron |  |  |
| rs201900069 | X:15612969 | C/T | T: < 0.001 | missense ~splice region | R/Q | 115 |
| rs199804629 | X:15618918 | C/T | T: < 0.001 | synonymous | L | 39 |
| rs768558803 | X:15618927 | G/A | A: < 0.001 | synonymous | A | 36 |
| rs761614932 | X:15618960 | G/A | A: < 0.001 | synonymous | A | 25 |
| rs73635825 | X:15618980 | A/G | G: < 0.001 | missense | S/P | 19 |
| rs765966061 | X:15619140 | G/T | T: < 0.001 | splice region ~intron |  |  |
